# Comparison of type II diabetes mellitus outpatient care cost by level of care

**DOI:** 10.1186/1471-2458-14-S1-P1

**Published:** 2014-01-29

**Authors:** Wan Norlida Ibrahim

**Affiliations:** 1 United Nations University, 56000 Cheras, Kuala Lumpur, Malaysia

## Background

Patients with diabetes often seek treatment and have follow-up as outpatients, be it at private or government health facilities. Data on economic burden of outpatient care are limited. Moreover, comparisons of costs for different outpatient settings are rarely done. The objective of this study was to compare the outpatient care costs for diabetes between primary and tertiary care level diabetes clinics.

## Materials and methods

This was a cross-sectional prevalence-based, retrospective cost of illness study, measuring economic burden of type II diabetes outpatient care in Malaysia using top down approach for provider cost and micro-costing for patient cost. The study population was all registered patients during the study period. It was conducted at selected diabetes clinics of government health facilities grouped by geographical regions.

## Results

A total of 489 patients from 10 centres took part in the study. Respondents were from 5 out of 11 states of Malaysia. Analysis of direct medical cost, direct non-medical cost and indirect cost showed significant difference between primary and tertiary levels. Direct medical provider cost was almost 7 times higher at tertiary care, direct medical patient cost was three times higher, direct non-medical and indirect cost were doubled. Annually every patient spent RM139.46 and RM438.46 for primary and tertiary level outpatient care, respectively. It costed the Ministry of Health Malaysia RM393.24 per patient per year to provide outpatient care at primary health clinics and RM2707.44 at tertiary diabetes clinics. The indirect costs were RM63.14 and RM147.77 per patient per year at primary and tertiary diabetes clinics, respectively.

## Conclusions

Cost for providing outpatient care for type II diabetes was significantly higher at tertiary care. Cost-effectiveness study of treating type II diabetes comparing both levels will justify the need for change of current practice in delivering diabetes follow-up care to contain cost.

